# An interpretative phenomenological analysis of the development and maintenance of gluten‐related distress and unhelpful eating and lifestyle patterns in coeliac disease

**DOI:** 10.1111/bjhp.12588

**Published:** 2022-02-15

**Authors:** Rose‐Marie Satherley, Fiona Lerigo, Suzanne Higgs, Ruth Howard

**Affiliations:** ^1^ Department of Psychological Interventions, School of Psychology University of Surrey Guildford UK; ^2^ Division of Population Health, Health Services Research & Primary Care University of Manchester UK; ^3^ School of Psychology University of Birmingham Edgbaston UK

**Keywords:** anxiety, coeliac disease, disordered eating, gastrointestinal disease

## Abstract

**Objective:**

Estimates indicate that individuals with coeliac disease are more likely to experience disordered eating and impaired well‐being than healthy controls, but less is known about the mechanisms by which these factors are related. The aim of this study was to understand experiences of coeliac disease and influence on subsequent unhelpful eating and lifestyle patterns.

**Methods:**

An online focus group discussion, hosted through a synchronous chat log, with adults living with coeliac disease was conducted. Seven individuals discussed their condition, lifestyle, and dietary changes post‐diagnosis. Discussions were analysed using an interpretative phenomenological approach, a technique that enables new practical or research insight into health conditions based upon participants’ experiences of their condition.

**Results:**

Three themes were identified: (i) Nobody knew what was happening to my body; (ii) I am so afraid of being ‘glutened’ that it is central to my thoughts and anxieties; and (iii) I am frightened but I can keep myself safe by being a ‘good’ coeliac. These appeared to contribute to participant distress or unhelpful eating or lifestyle behaviours. Participants appeared to develop severe anxiety around gluten, and implausible beliefs around diet and lifestyle management that appear to initiate and maintain unhelpful eating behaviours and maladaptive lifestyles changes, that contribute to distress.

**Conclusions:**

Extending current knowledge, we propose a novel cognitive perspective on the development and maintenance of disordered eating in coeliac disease. Implications for how health providers can better support individuals with coeliac disease, and the role of dietary management, anxiety, and gastrointestinal symptoms in the development of disordered eating are discussed.

**Statement of contribution**

***What is Already Known on This Subject?***

- Those with coeliac disease must maintain a strict gluten‐free diet, requiring increased control around food.
- For some, this dietary management appears to result in unhelpful, or disordered eating.
- This relationship is established, but the mechanisms are not well understood.

***What Does This Study Add?***

- Outlines two processes that elaborate established relationships between disordered eating and coeliac disease.
- Proposes a novel cognitive perspective on the development and maintenance of disordered eating in coeliac disease.
- Suggests intervention targets for addressing disordered eating in coeliac disease.

## Background

Coeliac disease is an autoimmune condition in which consumption of gluten, a protein present in wheat, barley, and rye, causes intestinal damage (Lebwohl, Sanders, & Green, 2018). Untreated, coeliac disease can lead to health complications including osteoporosis, lymphatic cancers, and cardiovascular disease (Green et al., 2003; Meyer, Stavropolous, Diamond, Shane, & Green, 2001). Current guidelines for management centre on a life‐long gluten‐free diet, which prevents consumption of gluten, reducing the symptoms and complications of coeliac disease (Lebwohl et al., 2013). Whilst ingestion of gluten is associated with symptoms and complications, simply touching gluten will not harm an individual with coeliac disease (Hall, Shaoul, & Day, 2020).

Gluten‐free diet maintenance is demanding, requiring increased monitoring and control around food, and avoidance of unsafe foods (King, Kaplan, & Godley, 2019; Silvester, Weiten, Graff, Walker, & Duerksen, 2016). Gluten avoidance is challenging, even consumption of trace amounts of gluten can affect individuals with coeliac disease (Cohen, Day, & Shaoul, 2019). Cross‐contamination is particularly common in social settings and can also occur when the same utensils are used to serve gluten‐containing and gluten‐free foods; however, using and washing plastic (over wooden) utensils thoroughly with soap and water is an effective way to prevent cross‐contamination (McDonald & Kupfer, 2020). The gluten‐free diet can lead to, or exacerbate underlying tendencies for, anxiety, depression, and impaired quality of life (Clappison, Hadjivassiliou, & Zis, 2020; Lebwohl et al., 2020; Liester & Liester, 2017).

Recently, psychosocial research has focussed on the development of disordered eating in coeliac disease. Whilst effective management of the gluten‐free diet requires vigilance around food, and gluten‐avoidance, some individuals may develop unhelpful eating behaviours, which impair psychological health and quality of life. In their formative model, Satherley, Howard, and Higgs (2015) suggest that whilst most appear to adapt well to a gluten‐free diet, strict dietary adherence and concerns around cross‐contamination may lead to limited food choices or eating behaviours, which may mimic or develop into disordered eating. Furthermore, Cadenhead et al. (2019) found that 53.3% of adolescents with coeliac disease described approaches to maintaining a gluten‐free diet, that included known risk factors for disordered eating (e.g., expressed rigidity, avoidance, controlling behaviour, and food preoccupation).

Despite work highlighting the relationship between gluten‐free dietary management and disordered eating, these relationships are not well understood. The aim of this study was to understand the experiences of adult participants with coeliac disease and what they believe contributes to the onset and maintenance of unhelpful eating and lifestyle patterns related to their disease management.

## Methods

This study uses Interpretative Phenomenological Analysis (IPA), a rigorous ideographic and phenomenological approach that seeks to generate contextualised knowledge from the individual’s perspective, to generate novel insights into patient experience (Smith, Flower, & Larkin, 2009). The data presented here were derived from an online focus group of adults with coeliac disease, in which participants were invited to type responses to questions in a synchronous chat log. Although interviews are typically used to gather data for IPA, when handled appropriately focus group data is suitable (Palmer, Larkin, de Visser, & Fadden, 2010; Randazzo, Farmer, & Lamb, 2015; Tomkins & Eatough, 2010). Here, the focus group method was used to replicate the forum‐style often used for information‐seeking and support by individuals with coeliac disease and allow access to a greater depth of insight into individual and shared life‐worlds. The resulting rapport and empathy within the group gave an experiential account suited to IPA as the group dynamic appeared to enable disclosures, and depth within the shared account that may not otherwise have emerged, whilst allowing individual accounts to be retained (Wilkinson & Smith, 2003).

### Participants

Participants for this study, were recruited as a purposive sample from a larger mixed methods project, in which participants (18–69 years) were invited through coeliac disease support groups, to take part in a study on eating behaviours, well‐being and lifestyle. This primary dataset included information on participants' willingness to try new foods, as assessed by the Food Neophobia Scale (FNS; (Pliner & Hobden, 1992)). The FNS is a 10‐item scale that has been validated in the general population but used more recently in adults with coeliac disease (Zysk, Glabska, & Guzek, 2019); scores above 35 are considered high, with lower scores indicating greater willingness to try unfamiliar foods.

For this study, participants who self‐reported a biopsy‐confirmed diagnosis of coeliac disease for at least 2 years and scored high on the FNS (above 35) were invited to take part in a focus group exploring the impact of their gluten‐free diet on food beliefs, eating behaviours, and well‐being. Those with suspected refractory coeliac disease, and additional food allergies or intolerances were excluded.

### Focus group content

The lead author facilitated the focus group, guiding but not directing conversation, through pre‐established questions and probes identified through a review of the literature addressing disordered eating in gastrointestinal disease (Satherley et al., 2015). Participants were asked about their symptoms and lives prior to coeliac disease diagnosis, followed by experiences at, and post‐diagnosis. Subsequent questions related to participants' experiences of coeliac disease in relation to their eating habits and well‐being, thoughts and behaviours, and changes in this over time (the focus group topic guide can be found in Table S1).

### Data collection

Participants were provided with an information sheet outlining the study. The first author contacted participants via phone to arrange a time and date to attend the focus group. Verbal consent was obtained via phone. Electronic, written consent was obtained before participants could join the password‐protected focus group, which was hosted through a secure online platform. The focus group was hosted as a synchronous, live discussion lasting 120 minutes. Participants were provided with a unique, non‐identifying username. The facilitator led group discussions by introducing questions and prompts. Participants typed their answers in response, and these were displayed on‐screen as a chat log, for others to read and respond to.

Ethical approval was obtained from the University of Birmingham research ethics committee. To enhance scientific rigour, this work had been conducted and presented in line with the JARS‐QUAL reporting standards (Levitt et al., 2018).

### Analysis

The focus group chat log was captured verbatim, within the web‐based software. Accounts were read and re‐read to ensure familiarity with the data. Guided by Palmer et al.'s approach (Palmer et al., 2010), the chat log was hand‐coded descriptively, line‐by‐line, with analysis focussed on keywords, descriptive and linguistic features of participant contributions, as well as conversational dynamics that appeared relevant to the origin of participants’ beliefs. Individual cases were coded before importing the data into NVivo (version 11) software, to facilitate organisation, systematic cross‐case coding, and theoretical interpretation of superordinate themes to identify and highlight meaning and experiences.

The chat log was coded separately by two researchers. Individual accounts and emerging group dynamics were coded (Smith, 2004). To ensure that coding represented the breadth or participant’s views adequately, coding was guided by the research questions but also by topics outside the research question. The coding was then checked for agreement and to identify heterogeneous and discrepant cases. Emergent codes were viewed by all authors for verification and to incorporate alternative understandings of the data. Researcher’s views and assumptions were noted as an integral part of the analysis process. The extracts presented here were selected to be typical of those expressed, or to provide the most powerful or insightful expression of presented themes. To preserve participant anonymity, all presented usernames are pseudonyms.

## Results

### Participants

Participants included seven adults (25–49 years) with self‐reported coeliac disease; all reported strict adherence to a gluten‐free diet for 3–13 years (Table 1). It was beyond the scope of this paper to present the types of disordered eating present in coeliac disease; these behaviours have previously been reported across a range of gastrointestinal diseases (e.g., see Cadenhead et al., 2019; Satherley, Higgs, & Howard, 2017, and Leffler, Dennis, Edwards George, & Kelly, 2007). Reported changes in eating behaviours focussed on food avoidance and appeared to mimic the Diagnostic and Statistical Manual for Mental Disorders (DSM) criteria for disordered eating, specifically Avoidant/Restrictive Food Intake Disorder (American Psychiatric Publishing & Inc, 2013). In some cases, food was avoided for days at a time, particularly when outside the home, as Tiffany explains ‘*I actually got to the point where I went to work dinners, birthdays, Christmas dinners, and didn’t eat anything. And we went on a girl’s holiday last year. I didn’t eat for days ‘cus of all the croissants and pastries they ate in the villa. I was starving!’* Participants also reported refusing to eat food prepared by others and refusing to attend environments where food was present (e.g., birthday celebrations).

**Table 1:** Overview of Participants

Participant Username	Gender	Age (years)	Diagnosis History	Years with Coeliac Disease Diagnosis
Michael	Male	47	‘*Years with stomach pains*’ and mouth ulcers prior to diagnosis. Michael thought he had cancer and was subsequently was diagnosed with irritable bowel syndrome and B12 deficiency prior to coeliac disease.	12
Hannah	Female	34	‘*Fobbed off with I(rritable) B(owel) S(yndrome)*’ diagnosis for ‘*several years*’. Repeated visits to the GP and private healthcare. Started a gluten‐free diet and felt better, and subsequently sought a biopsy confirmed coeliac disease diagnosis.	3
Debbie	Female	49	Described gastrointestinal symptoms to her General Practitioner and was referred to hospital for a biopsy confirmed coeliac disease diagnosis.	7
Fahdah	Female	29	Treated for irritable bowel syndrome 12 years prior to coeliac disease diagnosis, and referred onto the cancer pathway,. Experiences ongoing gastrointestinal symptoms. After repeat visits to the GP, Fahdah was referred to the hospital for a biopsy confirmed coeliac disease diagnosis.	13
Poppy	Female	31	After debilitating gastrointestinal symptoms and no diagnosis, Poppy cut out wheat and dairy from her diet. ‘*Several years later’,* she was referred to hospital for a biopsy confirmed diagnosis of coeliac disease.	4
Jade	Female	36	Delayed seeking care as she was fearful of needles. Overtime, symptoms escalated, and Jade had ‘*quite severe depression*’. She felt dismissed and misunderstood until she was referred on to receive a biopsy and coeliac disease diagnosis.	9
Tiffany	Female	25	Described debilitating gastrointestinal symptoms for years. Supported by her brother to seek coeliac disease diagnosis, but also received tests for cancer. Referred on to receive a biopsy confirmed coeliac disease diagnosis.	4

To establish the context for participant accounts, we present participants’ reflections on their experiences around their diagnosis, followed by accounts focussing on how dietary thoughts and behaviours develop and are maintained, and the impact of these on participants. What became obvious was that participants saw the impact of diagnosis and living with coeliac disease as a series of processes that evolve over time, as characterised in their narrative, storied accounts. Our analysis sought to capture these understandings through three overarching themes, each made up of subthemes that reflected the distinctive qualities for each participant (Table 2). Each overarching theme will now be described, illustrated with quotes from the chat log; subthemes, which contribute to these overarching themes, are highlighted in **bold** throughout the text. Exemplary excerpts and analytical insights for each overarching theme may be found in supplementary files; we highly recommend these files are read in conjunction with the following analysis.

**Table 2:** Overview of Overarching Themes and Subthemes, Each theme is further described within corresponding supplementary files

Overarching Theme	Subthemes	Illustrative Quote
1: Nobody Knew What was Happening to my Body (Table S2)	Afraid of what my symptoms might ‘really’ mean	I was so sick, wondering what was really going on. I was convinced I had cancer. I was scared and saying my last prayers. *Michael*.
	‘I Wasn’t Believed’	The GP kind of fobbed me off for quite a few years, I wasn’t believed, they didn’t believe me. *Fahdah*
	Searching everywhere for ways to avoid ‘getting glutened’	I’ve also read this on the internet… Anyone else dealing with this? This gets overwhelming, I keep finding out more CC [cross contamination] dangers. *Hannah*
2: I am so Afraid of Being ‘Glutened’ that it is Central to My Thoughts and Anxieties (Table S3)	Feeling ‘Traumatised’ by this Disease	My body was in so much pain and I was so scared, I don’t want to be sick forever more. I have like this PTSD trauma lol [laugh out loud]. *Jade*
	Preoccupied with Gluten avoidance	I see breadcrumbs everywhere! I think of all the ways cross contamination can occur. There is a part of me that wonders if I am succumbing to some kind of collective gluten paranoia. *Tiffany*.
3: I’m Frightened but I can Keep Myself Safe by Being a ‘Good’ Coeliac (Table S4)	Managing my Gluten‐Free Diet Well	If you want to know for certain that your food is safe, you need a gluten‐free kitchen or very strict separation/limit gluten in your kitchen altogether. We have one counter devoted for the 'wheat eaters' because I can't buy gluten‐free bread for all… they [my family] know the drill. Butter and jam containers are labelled strictly to maintain gluten‐free. *Poppy*
	Maintain a Sense of ‘Control’	I won't even allow gluten products in my home, nothing not toiletries or washing powder, not glues or paints, not pet foods or litters. I gave up trying control restaurants long ago. *Hannah*
	Fear of Gluten I Can’t See	Some things that have caused my gluten rashes include those blood pressure bands at the doctor's, shared pens (like sign‐in sheets), suitcases in my cupboard from my ‘pre‐Gluten‐Free’ days, or unwashed new clothes, my keys after my son borrows the car. *Debbie*
	Explanations of Routes to Cross‐Contamination	well, let's get real here‐‐‐if it [kissing] involves swapping spit‐‐well, yes, it is a problem. Without getting too ‘icky’‐‐there is mucosa in your mouth lining and swallowing…etc, etc. *Jade*

### Theme 1: Nobody knew what was happening to my body

Participants expressed that they were **Afraid of What My Symptoms Might ‘Really’ Mean** for a long time prior to receiving a diagnosis. Receipts of incorrect diagnoses were common, including both Tiffany and Fahdah who were misdiagnosed with life‐threatening diseases (cancer). Accounts were accompanied by feelings of anger and frustration towards health providers. As Jade says, ‘*no one understood, everyone judged. Even the doctors … I’d had various tests from the hospital, and nothing came up’*. The failure to quickly, and accurately, identify the cause for their symptoms, and in some cases perceived health provider reluctance to engage respectfully with participants’ discomfort and ill‐health, left most participants feeling unsupported and questioning their experiences. Fahdah summarizes this experience, ‘***I Wasn’t Believed***’, and Jade explains, ‘*I was beginning to think I was a bit of a hypochondriac really’*.

Participants’ felt unprepared for effective living post‐diagnosis and unsupported by clinicians. Therefore, advice was sought from friends, family, or online forums, with participants **Searching for Ways to Avoid ‘Getting Glutened’**. This is evident in the dialogue of participants, which is stylised and includes new terminology, (e.g., ‘*CC’d’* (cross‐contaminated) and *‘glutened’),* to refer to instances of gluten exposure. All participants appeared to be familiar with these new terms. For example, when the forum discussion turns to kissing Jade says ‘*Let's get real here‐‐‐if it involves swapping spit‐‐well, yes, it is a problem. Without getting too "icky"‐‐there is mucosa in your mouth lining and swallowing*’ and Debbie says ‘*I did a bit of googling and found out that the stitches* [in her plaster cast] *contained gluten in them*.’ These sources of information appeared to result in misinformation about potential sources of gluten cross‐contamination.

### Theme 2: I am so Afraid of Being ‘Glutened’ that it is Central to My Thoughts and Anxieties

As described by authors elsewhere (Rose & Howard, 2014; Taylor, Dickson‐Swift, & Anderson, 2013), being diagnosed with coeliac disease changed how participants thought and felt. However, our participants go beyond this, reporting **Feeling ‘Traumatised’ by this Disease**. Four spoke of persistent and vivid memories, intrusive flashbacks, or recurring dreams associated with gastrointestinal symptoms and gluten exposure. Poppy reports a vivid memory around gluten exposure, comparing the clarity of her memory to ‘*Princess Diana’s death*’, and remembering details of the clothing she wore at the time of her symptoms. Marked alterations in arousal and reactivity were evident. For example, in Jade’s description of a nightmare in which she ate gluten; *‘I woke up in a complete panic and was so worked up I couldn't get back to sleep. My heart was racing, I was sweating, I was breathing fast’*. In addition, three participants spoke of a sense of dissociation from their bodies, and one spoke of angry outbursts related to gluten exposure (see Table S2).

Central to most accounts is being **Preoccupied with Gluten Avoidance**, as Tiffany says ‘*I see breadcrumbs everywhere! I think of all the ways cross contamination can occur’*. Collectively, participants felt overwhelmed and distressed at the thought of re‐experiencing gastrointestinal symptoms in response to accidental gluten exposure. Fahdah says of a time where she suspected she had unintentionally consumed gluten, ‘*I remember pacing up and down that carpet, fizzing all over my limbs and in my head, like a tingling’*. To avoid gluten‐exposure, participants restrict their lifestyle in ways that impact their quality of life and feel a sense of loss related to both the inability to take part in valued activities that were central to their quality of life, and to a loss of parts of their personality that they feel characterised who they were prior to coeliac disease diagnosis. For example, Michael says *‘I only really eat at home, away from other food. Gluten free, cooked by me. It has made long shifts or work trips hard. I can now go a few days without if I need to, it’s very unfair’*.

### Theme 3: I am Frightened but I can Keep Myself Safe by Being a ‘Good’ Coeliac

All participants described behaviour and views consistent with current medical and dietary guidelines, a subtheme we entitled: **Managing My Gluten‐Free Diet Well**. This included adaptations to diets that reduced the risk of gluten consumption (e.g., asking about food preparation, carrying gluten‐free foods, adopting reasonable caution when eating outside the home). This is summarized by Debbie who says, ‘*I stay clear of gluten, explain, explain, explain to everyone, and always carry snacks with me!’* To reduce concerns around gluten contamination, some attempted to **Maintain a Sense of ‘Control’** in environments in which their food was prepared. Control in the home took many forms, sometimes this control appeared helpful, for example, cleaning, using separate utensils for gluten‐free and gluten‐containing foods, and segregating kitchens into gluten‐free and gluten‐containing zones. Participants also reported restrictions to the eating, food preparation, and other activities of those with whom they live, for example, by maintaining a gluten‐free household (e.g., gluten‐free food, toiletries, and furnishings). As Hannah explains ‘*Only gluten‐free soaps and shampoos for me and my children. And even hair dye. It's just not worth the risk*’. Outside the home, participants avoided areas where they thought they might be exposed to gluten, including avoiding social events, supermarkets, holidays, and travel. Several reported separating their external environments into ‘*safe*’ and ‘*unsafe*’ zones or planning ahead in an attempt to gain control over unfamiliar environments. This can be functional, for example, Debbie says in her account of the controls used to ensure her workplace, a bakery, met her needs: ‘*I work shorter shifts now so my exposure is reduced. I clean obsessively. I have special soap that removes the gluten, I have my special mask. It’s so strict. But if my boss was to ever go, I would be stuck. She lets me control everything, clean it all, have it my way’*. Control here can be contrasted with the kind of control‐seeking that is represented in the views presented below, which appears to impact quality of life.

A **Fear of Gluten I Cannot See,** was an experience central across participant accounts. Here, participants demonstrate the consistency with which they held beliefs about potential sources of gluten cross‐contamination, which when compared to research and clinical guidelines for gluten‐free dietary management, the research team believed were implausible (Hall et al., 2020; Lebwohl et al., 2018). These fears focussed on potential sources of cross‐contamination other than ingestion, including airborne and contact contamination with gluten‐containing products. This type of thinking was prevalent, with 68 separate references made in the focus group, constituting over 35% of the total content. These beliefs appear to be organised around two focal points. Firstly, anxiety about avoiding contamination and the extrapolation of dietary gluten‐avoidance measures to other everyday products. For example, Hannah states: ‘*I don’t think it’s just about prepping food though. I don't know if my soap and hair products have gluten in them’*.

Secondly, participants appear to relate to a fear of very tiny particles of gluten, which we have termed ‘*micro‐gluten’*. These include references to ‘*crumbs*’ or ‘*specks*’ of gluten. The fear is that these particles will result in cross‐contamination, and thus illness. These contaminants go unseen (i.e., they are in food prepared by others or are transferred from contaminated work areas, eating utensils, or cookware, or are too small to be seen), and can be passed to the person with coeliac disease via implausible pathways, such as respiratory transfer, skin contact or being biologically self‐generated by the participant. Tiffany says*: ‘I read on the internet about this thing called the ketone diet… it can be dangerous for coeliacs because when your body produces ketones, it burns the fat muscles which contain gluten – so can you like internally gluten yourself?’*

Participants wished to provide **Explanations of Routes to Cross‐Contamination** to the researcher and other participants. When identifying sources of cross‐contamination, Tiffany describes retracing her steps and identifying her make‐up as ‘*the clear culprit’* of her symptoms. However, whilst justifying their beliefs the holder sometimes simultaneously appears aware of its implausibility, querying whether they are acting ‘*paranoid*’ or ‘*over the top*’. These beliefs, whilst containing some truth, appear both implausible to others and, for some, resistant to challenge. Debbie describes her concerns about experiencing contamination from wallpaper paste, Michael responds ‘*But the wallpaper paste is glued to the wall? It can’t get into the air? So you shouldn’t be able to breathe it in? Unless you’re eating it?*’, Debbie resists the challenge to this belief stating ‘*To me it’s not safe. It is gluten’*. Participants evidence these beliefs by using their own experience, ill‐reasoned science/medicine‐based evidence, and other people’s opinions. In summary, these beliefs and explanations appear to be distressing, preoccupy the holder, and be held with some certainty, as evidenced by their resistance to challenge by other participants.

## Discussion

Whilst previous work (Satherley et al., 2015) has described relationships between gastrointestinal disease and disordered eating, this analysis provides insight into these relationships and gives an account of the lived experience of individuals who reported unhelpful eating and lifestyle behaviours due to following a gluten‐free diet. This paper details three novel contributions: it outlines experiences and behaviours that participants feel impair their quality of life, finds that patients well‐being could be impaired despite their maintaining what might appear clinically to be ‘good adherence’ to a gluten‐free diet, and describes apparently maladaptive coping strategies due to dietary adjustments.

Established cognitive formulations (Beck, Emery, & Greenberg, 2005) highlight the role of irrational beliefs in many types of disordered behaviour, and recent work demonstrates the role of gastrointestinal‐specific beliefs in the development of disordered eating (Murray et al., 2020). Concordant with Satherley et al.’s (2015) model, it was evident in this analysis, that fear of re‐experiencing pre‐ and post‐diagnosis symptoms, which were often gastrointestinal, contributes to the development of unhelpful or implausible beliefs around cross‐contamination and subsequent anxiety, distress, and eating behaviours that appeared disordered. Extending this model, this analysis indicates that these beliefs, alongside the high levels of reported anxiety, may be similar to other kinds of irrational beliefs formation detailed within cognitive formulations, having qualities of certainty, distress, and preoccupation (Green et al., 2008). According to these formulations, the presence of irrational beliefs biases information processing towards certain types of information that reaffirm beliefs; for our participants, interpretations of their bodily sensations were attributed to sources of potential gluten exposure. Here, it is plausible that the discomfort and fear caused by pre‐diagnosis gastrointestinal symptoms, the challenges and time associated with obtaining a conclusive diagnosis, the fear of re‐experiencing symptoms, and living with the changes required by a gluten‐free diet result in coeliac‐specific irrational beliefs for some individuals. However, the core components of these beliefs (i.e., vigilance and preoccupation with gluten) are essential to gluten‐free diet management and may function to protect individuals from gluten exposure.

Extending previous work (Satherley et al., 2015) that hypothesized the role for dysfunctional illness beliefs around food, namely the belief that all foods have cross‐contamination potential, this analysis uncovers the processes through which these beliefs may develop. Perceiving limited clinical support post‐diagnosis, and with limited evidence to understand cross‐contamination fears, our participants appeared to elaborate upon the available evidence, using sources such as internet support‐groups to obtain information. Without accurate and complete information about gluten cross‐contamination, subsequent sense‐making may result in erroneous explanations for symptoms. For example, participant explanations for symptoms included gluten exposure from non‐food products, supermarkets, and animals. As described by Gunn and Larkin (2020), these explanations for symptoms may function to help individuals make sense of alterations to their body, and these painful and distressing experiences.

The experiences outlined here, that may relate to the onset and maintenance of disordered eating in coeliac disease, have much in common with processes that maintain other kinds of disorder characterised by an erroneous belief system. The Threat Belief Model (Freeman et al., 2004; Freeman, Garety, Kuipers, Fowler, & Bebbington, 2002) suggests that erroneous beliefs arise to make sense of events, and that multiple factors, including affective processes and anomalous experiences contribute to erroneous belief formation. Freeman ( 2007) suggests that anxiety is a key contributory factor, that internal and external events are interpreted in line with the current emotional state, and that erroneous belief formation often occurs during situations of emotional distress. Of key importance to the fit of this model with both the analytic method used here (IPA) and participant’s unhelpful beliefs, is the notion that it is the person's search for meaning and selection of an explanation that drives erroneous belief formation. By using IPA, researchers could access the participants’ own meaning‐making and explanation, thus making it possible to map the analysis to a theorised pathway by which these might lead to erroneous belief formation that appears to contribute to distress and unhelpful eating behaviour.

Furthermore, Freeman et al. (2002), Freeman et al. (2004), and Freeman (2007) suggest that heightened anxiety plays a role in *catastrophising*, a cognition which has been suggested to contribute to disordered eating in children with gastro‐intestinal disease (Newton, Schosheim, Patel, Chitkara, & van Tilburg, 2019). Freeman et al., further suggest that safety behaviours and continued levels of heightened anxiety are the principal contributors to erroneous belief maintenance. This analysis suggests that unhelpful eating patterns, or disordered eating, may be related to erroneous reasoning and persistent anxiety. We hypothesize that both the content of beliefs and anxiety processes appear to be involved in maintaining disordered eating.

To manage anxiety related to disease symptoms, our participants adopt eating and lifestyle strategies that may function as safety behaviours (Clark & Steer, 1996; Salkovskis, 1991). Similar behaviours have been identified across gastrointestinal diseases, including limiting activity and social events (Windgassen, Moss‐Morris, Goldsmith, & Chalder, 2019). Cognitive models suggest that safety behaviours play a role in the maintenance of anxiety (Clark & Steer, 1996), and delusional belief (Freeman et al., 2007). It is plausible that the behaviours described by the participants in this study, (e.g., repeated hand cleaning, avoidance of social events involving food), which are perceived by participants as affording protection from gluten exposure, serve as safety behaviours that escalate anxiety and thus maintain implausible beliefs, distress, and eating behaviours that appear disordered. Four types of safety behaviour emerged from our analysis: *avoidance* (e.g., not attending family events, only eating food they have made); *hypervigilant focus*, (e.g., mental rehearsal to avoid sources of contamination); *ritual actions*, (e.g., zoning food preparation areas, checking non‐food items for gluten contamination); and *help*, (e.g., seeking information and support from online forums, and clinicians). For our participants, these behaviours typically increased after instances of gluten exposure.

**Figure 1:**
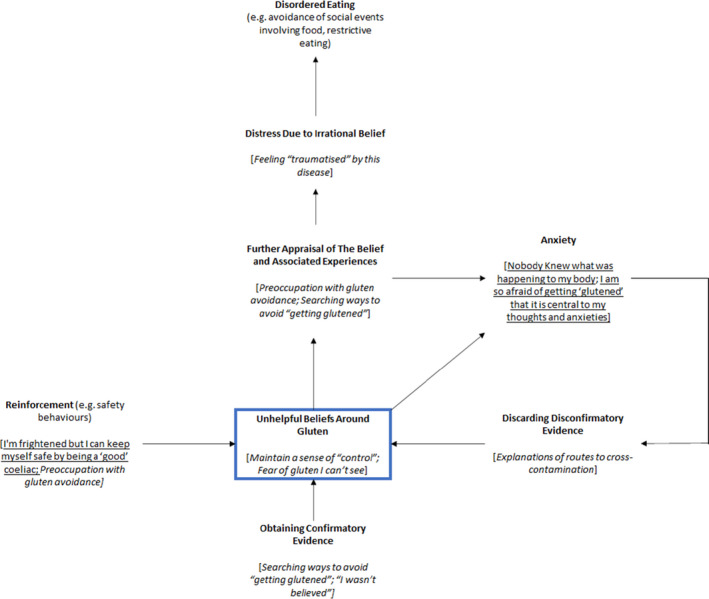
Development and Maintenance of Gluten‐Related Distress and Unhelpful Eating and Lifestyle Patterns. This framework is adapted from Freeman et al.’s cognitive model of belief maintenance (Freeman, 2007). The themes resulting from the present analysis are mapped onto this model, to provide a framework for the development and maintenance of gluten‐related distress and unhelpful eating and lifestyle patterns in coeliac disease. Underlined text represents overarching themes from the analysis, and *italicised text* represents sub themes.

In Figure 1, we depict a framework adapted from Freeman (2007), that maps the themes contained in the present participants' accounts onto this framework. This framework conceptualises unhelpful beliefs around gluten as threat beliefs. As in Freeman’s model (2007), we propose that threat beliefs are maintained by: (1) reinforcement, for example, safety behaviours (e.g., managing diet well), which give temporary relief from the perceived gluten‐related threats; (2) obtaining confirmatory evidence (e.g., searching peer support groups for ways to avoid ‘getting glutened’); and (3) discarding disconfirmatory evidence (e.g., in the group dynamic when ‘challenges’ to belief rationality by other group members were resisted). In line with Freeman’s conceptualization (Freeman, 2007), we also suggest that participants demonstrate further appraisal of these beliefs, (e.g., awareness, preoccupation, and justification/reasoning), and increased anxiety around ‘getting glutened’ with threat beliefs as the focus. Both of which we suggest lead to further distress, (e.g., feeling traumatised by this disease, and disordered behaviour, here what may be considered, disordered eating.

### Strengths and limitations

The core of this analysis focuses on unhelpful or implausible beliefs around gluten exposure. There are challenges in interpreting and classifying these beliefs as implausible; whilst no member checks were made when attributing irrationality to some of the ideas presented by participants, it is clear from the accounts that many participants believed themselves, and each other, to have ideas about gluten that were erroneous. However, consistent with the IPA approach, we do not rule out the possibility of alternative interpretations. Accordingly, the conclusions drawn are deliberately cautious and these types of belief warrant further exploration.

Participants were not diagnosed with disordered eating but were recruited because they had elevated scores on the FNS, indicating a fear of trying new foods, a key feature of Avoidant Restrictive Food Intake Disorder (American Psychiatric Publishing & Inc, 2013). Additionally, all participants reported eating behaviours that mirror DSM criteria for disordered eating, including behaviours to restrict and/or control food intake, which appeared to impair quality of life (American Psychiatric Publishing & Inc, 2013). Furthermore, the experience of this participant group represents only the experiences of a sub‐group of individuals with coeliac disease, who report impairments in quality of life and well‐being due to coeliac disease diagnosis and the need to follow a strict, gluten‐free diet.

### Clinical and research implications

This analysis highlights several potential intervention points to support quality of life and well‐being in coeliac disease, whilst maintaining a strict gluten‐free diet. There is a growing body of research that indicates that airborne or contact gluten contamination is unlikely (Hall et al., 2020), clear and unified guidance on management of gluten‐contact whilst following a gluten‐free diet has not been addressed. In line with Stahl et al., (Stahl, Mehta, Liu, & Shull, 2020), this work supports the need for evidence‐based guidelines for the gluten‐free diet, including rigorous assessment of gluten exposure from food and non‐food products.

Participants’ experiences of diagnostic error and delay contributed to heightened anxiety, the subsequent development of unhelpful dietary beliefs, and changes to dietary and lifestyle behaviours. Whilst not a formal diagnosis, several participants report experiences that appear consistent with a clinical or subclinical diagnosis of post‐traumatic stress disorder (PTSD; American Psychiatric Publishing & Inc, 2013). As has long been supported, our research reinforces the importance of the early identification and diagnosis of coeliac disease, and the provision of post‐diagnosis education.

These participants' experiences suggest that contact with health providers is an important factor in gluten‐related distress, and a focus of unhelpful beliefs that impact quality of life. Clinical support post‐diagnosis is essential in helping individuals develop confident management of the gluten‐free diet (Ludvigsson et al., 2014). Whilst evidence‐based interventions to address unhelpful beliefs and eating patterns in coeliac disease have not yet been developed, this formative evidence, alongside the increasing work addressing psychological interventions in other gastrointestinal diseases (Kinsiinger, 2017) suggests a number of ways that healthcare providers might further support those with coeliac disease. Firstly, clinicians should not assume that patients who appear to have good gluten‐free dietary management do not have reduced well‐being. Secondly, upon diagnosis, sources of accurate information and support, especially around cross‐contamination should be emphasized. Psychoeducation could focus on education around the gluten‐free diet, the psychological impact of coeliac disease, dispelling myths around cross‐contamination, and identifying reliable sources of support. Thirdly, referral to psychological support is critical for identifying, assessing, and addressing impaired well‐being and unhelpful thoughts and behaviours around food within this population. Given the success of psychological intervention in other gastrointestinal diseases, we tentatively suggest that intervention may include cognitive restructuring to address symptom‐related anxiety and hypervigilance, problem‐ solving skills, and exposure techniques to address safety behaviours that serve to reinforce unhelpful beliefs (Kinsiinger, 2017).

This work has wider research implications and offers the opportunity to advance our understanding of disordered eating. Firstly, additional research should establish if airborne and contact are indeed sources of gluten contamination, and if so, what levels of gluten are potentially harmful for individuals with coeliac disease. Secondly, coeliac disease may serve as a discrete tracer condition within which a greater understanding of other kinds of disordered eating might develop. To further our understanding, experiences of those with both a confirmed diagnosis of disordered eating and coeliac disease is required. Finally, future research should focus on interrelationships between disordered eating symptoms and anxiety. The onset of coeliac disease may form a specific time‐bound set of environmental and biopsychosocial conditions within which disordered eating is established, providing a framework within which to explore theoretical explanations for the development and maintenance of disordered eating, and intersections with gastrointestinal disease.

## Author contribution

**Rose‐Marie Satherley:** Conceptualisation; Data curation; Formal analysis; Investigation; Methodology; Project administration; Resources; Software; Validation; Visualisation; Writing – original draft; Writing – review & editing. **Fiona Lerigo:** Data curation; Formal analysis; Software; Validation; Visualisation; Writing – original draft; Writing – review & editing. **Suzanne Higgs:** Conceptualisation; Methodology; Supervision; Validation; Writing – review & editing. **Ruth Howard:** Methodology; Supervision; Validation; Writing – review & editing.

## Conflict of interest

All authors declare no conflict of interest.

## Ethical approval

Ethical approval was obtained from the University of Birmingham Science, Technology Engineering and Mathematics Ethical Review Committee (Application Number: ERN_15‐0370A).

## Supporting information


**Table S1**. Focus group guide.

**Table S2**. Theme 1 ‐ Nobody Knew What was Happening to My Body.
**Table S3**. Theme 2 – I am so Afraid of Being ‘Glutened’ that it is Central to My Thoughts and Anxieties.
**Table S4**. Theme 3 – I’m Frightened but I can Keep Myself Safe by Being a ‘Good’ Coeliac.

## Data Availability

The qualitative datasets analysed during this study are not publicly available due to ethical guidance around participant anonymity. Anonymized transcripts are available from the corresponding author on reasonable request.
