# Supplementary material for: An interpretative phenomenological analysis of the development and maintenance of gluten‐related distress and unhelpful eating and lifestyle patterns in coeliac disease
Source: Br J Health Psychol. 2022 Feb 15;27(3):1026–42. doi: 10.1111/bjhp.12588 (PMC9544439; doi:10.1111/bjhp.12588)
Supplement: Supplementary file 1 — Table S1. Focus group guide. [file BJHP-27-1026-s001.docx]

| **Example Questions** | **Prompts** |
| --- | --- |
| Could you tell me about your coeliac disease, and how you came to your coeliac disease diagnosis? | - Can you tell me about your symptoms before diagnosis? - How did arrive at your decision to seek support? - Did you face any challenges? What challenges did you experience? - How did you feel at this time? |
| Could you tell me about your gluten-free diet, and how you’ve managed this overtime? | - Did you make changes? What changes did you make? - Does anything make this more challenging/easier? - Can you tell me about what happens if you are exposed to gluten? - How does this make you feel? |
| How do you determine whether something contains gluten or not? | - How do you arrive at this decision? - What challenges do you face? - Can you tell me what happens when you’re unsure? |
| Can you tell me about a time or situation where you’ve felt concerned around your gluten-free diet? | - How long ago? - In what ways? - How did you feel at this time? |
| What has coeliac disease changed in your life, and in what way? | - Did you learn anything? - Did you make changes? - Would your life have been different if you had not been diagnosed with coeliac disease? |
| Have your experiences with coeliac disease and the gluten-free diet changed the way you think about food? How? | - Do you view food differently now than before you had coeliac disease? - In what ways? - How does this may you feel? |

**Supplementary File 1.** Focus Group Guide.
