# Supplementary material for: An interpretative phenomenological analysis of the development and maintenance of gluten‐related distress and unhelpful eating and lifestyle patterns in coeliac disease
Source: Br J Health Psychol. 2022 Feb 15;27(3):1026–42. doi: 10.1111/bjhp.12588 (PMC9544439; doi:10.1111/bjhp.12588)
Supplement: Supplementary file 2 — Table S2. Theme 1 ‐ Nobody Knew What was Happening to My Body. Table S3. Theme 2 – I am so Afraid of Being ‘Glutened’ that it is Central to My Thoughts and Anxieties. Table S4. Theme 3 – I’m Frightened but I can Keep Myself Safe by Being a ‘Good’ Coeliac. [file BJHP-27-1026-s002.docx]

| **Supplementary File 2: Theme 1 - Nobody Knew What was Happening to My Body**  Underlined text demonstrated instanced of stylised, Americanised or new terminology | | |
| --- | --- | --- |
| **Subtheme** | **Analysis** | **Illustrative Quotes** |
| Afraid of what my symptoms might ‘really’ mean | Misdiagnosed and unsupported (6 participants)  3 participants received incorrect diagnoses for irritable bowel syndrome.  Two participants were faced with alternative, life-threatening diagnoses. In both cases, participants were referred onto cancer testing services.  Failing to find a cause for their symptoms, 5 participants describe feeling unsupported by clinicians and alone in their experiences. | *I probably had some form of symptoms for several years and been investigated for various different things like I[rritable] B[owel] S[yndrome].* **Hannah**  *I went to the GP and asked for some blood tests for CD … and then she’d also put me in to have all sorts of tests for cancer… I was convinced I had cancer.* **Tiffany**  *I live in a quiet part of the world with not much understanding of coeliac… the doctors aren’t so good at CD diagnosis! It’s hard to identify and the doctors always think it’s something different.* **Michael** |
|  | Fearful of death (2 participants). | *I was terrified, really scared that I was dying. And we cancelled the holiday. I remember feeling devastated. I remember laying there, holding the carpet, feeling the hairs on the carpet, and thinking this is it. My life is over.* **Fahdah** |
| “I wasn’t believed” | Feeling dismissed and questioned (5 participants) | *The biggest problem I had was pain in my joints. The GP kind of fobbed me off for quite a few years, I wasn’t believed, they didn’t believe me.* **Fahdah**  Echoing this experience, **Hannah** responds: *Seems like a lot of people were fobbed off.* |
|  | Questioning symptoms (3 participants) | *No one understood, everyone judged. Even the doctors. … I’d had various tests from the hospital, and nothing came up. I was beginning to think I was a bit of a hypochondriac really.* **Jade**  *I felt like a bit of a fraud always going back and saying things like, I’m just really tired.* **Hannah** |
| Searching for ways to avoid “getting glutened” | Information Seeking (7 participants)  Online support groups were a key source of information. Interactions across the group reflected a jargonised coeliac ‘language’, and Americanised spellings were frequently used, which might indicate their source is US internet resources including forums for providing advice or information around coeliac disease.  This information seeking appeared to result in sources of misinformation. | *I’ve also read this on the internet… Anyone else dealing with this? This gets overwhelming, I keep finding out more CC dangers* **Hannah**  *I still get problems with CC at times, usually from baked foods. I’ve been glutened by them a few times.* **Debbie**  *If it involves swapping spit--well, yes, it is a problem. You will get glutened. Without getting too "icky"--there is mucosa in your mouth lining and swallowing ...etc* **Jade** |

**Supplementary File 3: Theme 2 – I am so Afraid of Being ‘Glutened’ that it is Central to My Thoughts and Anxieties**

| **Subtheme** | **Analysis** | **Illustrative Quotes** |
| --- | --- | --- |
| Feeling “Traumatised” by this disease | Vivid memories/reexperiencing dreams (3 participants)  Rather than dreams, two participants reported vivid memories in detail. These memories display a sense of time distortion, consistent with symptoms of a post-traumatic-like stress disorder. | *Last night I had a dream that I just kept eating "normal" pizza, and I was terrified that when I woke up it would be true and I would be sick for the next three days.* ***Michael***  *I forgot about the single gluten nightmare I had. I was at a fair and for some reason was wandering alone and meeting my husband somewhere. I was on my way to meet him and was starving. I passed by a hotdog stand and thought that sounds delicious! I had eaten half of one, before I remembered that hotdog doesn't mean gluten free. I woke up in a complete panic and was so worked up I couldn't get back to sleep. My heart was racing, I was sweating, I was breathing fast. Before I realised I was in bed I actually checked my mouth for food.* ***Jade***  *It’s like when Princess Dianna died, I remember where I was, what I was wearing, what I was doing. I can tell you now, I was wearing my red Fat Face t shirt when I was crying in pain. Funny how you remember the small things. I must have been so alert to everything around me. It’s crystal clear in my head. I can feel those symptoms like they happened yesterday.* **Poppy** |
|  | Dissociation (3 participants)  Participants describe a sense of dissociation, centred on disconnecting from their bodies due to ongoing pain. | *I remember… laying down and looking at the ceiling and thinking – this is it. What’s the point in being here? I’m trapped in a body that’s not mine.* **Fahdah**  *It’s almost worse now than it was before my diagnosis. My insides are turned upside down, my head becomes this cloud. You suddenly don’t remember who you are and the world goes on by without you.* **Michael** |
|  | Angry Outbursts Related to the Traumatic Experience (1 participant) | *I’m scared of cross contamination everywhere… The other day, my daughter walked in with a French stick. Right through my kitchen. I thought why on earth has she got that here, she knows it will poison me! I was out of my mind, screaming – don’t bring that poison in my house.* **Hannah**  **She continues:** *That made me so furious, and scared. I was petrified. All those crumbs dropping all over my clean floor. I’m going to have to clean those up, the breadcrumbs will get on my hands and ultimately in my mouth. It’s the little things like that, that people forget.* |
| Preoccupation with gluten avoidance | Preoccupation with Gluten Avoidance (7 Participants) | *I see breadcrumbs everywhere! I think of all the ways cross contamination can occur … When I moved into shared uni housing I thought I would lose my mind. Breadcrumbs would travel, I was worried about others eating toast, and using the toaster. I must have washed my hands 10 times every time I ate.* **Tiffany**  **Later she says:** *Double check soaps, shampoo. While not a direct issue, others are going to touch it, touch their hair, touch everything else.*  *With gluten free items, will I need to verify with the manufactures every time I buy a product to verify cross contamination has not taken place within their factory for my gluten free food?* **Tiffany** |
|  | Scared of Symptoms Returning (All Participants) | *I remember pacing up and down that carpet, fizzing all over my limbs**and in my head, like a tingling. And then laying down and looking at the ceiling and thinking – this is it. What’s the point in being here.* **Fahdah**  *I am completely terrified to eat anywhere but my home which I share with family members that eat gluten. I’m on edge all the time, alert, watching. The fear, it’s exhausting***. Hannah**  *I’m scared of feeling sick and tied again, always on edge… doing anything to not be back to where I was.* **Jade** |
|  | Identity Loss (All Participants) | *This is so much and I cannot help but feel like I'm losing a part of myself, I’ve lost some of who I am. That my life is isolated and everything I do is around preventing cross contamination.* **Hannah**  *I was a pastry chef for the first ten years, and that has been the hardest part of the diagnosis. I started baking at 6 years old, and then I went back to culinary school and got my culinary degree. And then being told I can’t cook the way I did, and I can’t bake the way I did was really hard… But I think that’s the hardest part. I think of the diagnosis, having to change my lifestyle in terms of career. What I’ve known for most of my life, that was the hardest part. It still is the hardest part.* **Fahdah** |

**Supplementary File 4: Theme 3 – I’m Frightened but I can Keep Myself Safe by Being a ‘Good’ Coeliac**

Underlined text demonstrates narratives around tiny particles of gluten, or micro-gluten’s

| **Subtheme** | **Analysis** | **Illustrative Quotes** |
| --- | --- | --- |
| Managing my Gluten-Free Diet Well | Dietary Changes (7 participants) | *I just remove all gluten from my diet. Not a crumb will pass my lips.* **Tiffany**  *I think of it like* *I can do this, I stay clear of gluten, no gluten at home. Explain, explain, explain to everyone - 100% gluten free only. I am only gluten free!* **Debbie** |
|  | Food Preparation (7 participants) | *I’m going to be doing some research in terms of the different flours I can use and to see what I can get. I’m racking up a nice little collection of gluten free books.* **Debbie**  *I only cook gluten-free now for our whole family; if they use bread, they know the drill. Butter and jam containers are labelled strictly to maintain gluten-free; breaded chicken and pizza are the only gluten-items allowed in the kitchen anymore and must be kept away from any other foods while out.* **Fahdah**  *We had to move to a dedicated gluten-free house, as my accidental exposures made me so unwell.* **Michael** |
|  | Reasonable Caution When Eating Outside the Home (7 participants)  Participants report a number of controls that centre around questioning those preparing food, carrying gluten-free food on their person, or eating at trusted restaurants. | *When I eat outside the home it’s only at the places I know are going to be 100% safe, coeliacUK accredited.* **Debbie**  *There’s an event this weekend and it’s a hog roast. But I’ve been in touch with the organisers who were very willing to buy me a gluten free roll but because of the cross contamination and they’re catering for 100 odd people, you guarantee anything. So I’m just taking my own food.* **Poppy**  *I always keep a box of nuts and fruit and a banana in my bag, that will always keep me going until I find something.* **Tiffany** |
| Maintain a Sense of “Control” | Use of control to manage anxiety and distress (7 participants)  Participants felt able to manage their anxiety around cross-contamination when implementing controls over their environment.  Some reported a need to maintain a gluten-free home, this extended beyond food, to household products (4 participants)  Attempts to control environments outside the home, ensured management of anxiety and distress. Control methods included repetitive cleaning and “*zoning*” of the environment, and obsession-like cleaning | *I’m safe in my family bubble where everyone understands the controls but as soon as you step outside your bubble, it’s a world of danger and unknown.* **Fahdah**  **Jade** responds: *I agree, a safe bubble, that you can control, and you can be safe in. In my bubble I can eat, I can socialise, I can relax. Outside it’s very different!*  *My main concern is the fact that I live in this house, where absolutely no one is supportive of me… I’m worried about others eating toast, and using the toaster… I started waking up at 3am so I could clean and cook before anyone else, and adjusted all my meal times accordingly.* **Tiffany**  *I’m having hardwood floors installed and have looked everywhere to see if there’s gluten in the product. I know spackle might contain gluten, so I’m very worried. Have called the manufacturers, asked how the floors are built and manufactured.* **Poppy**  *Only gluten-free soaps and shampoos for me and my children. And even hair dye. Its’ just not worth the risk.* **Hannah**  *I work shorter shifts now so my exposure is reduced. I clean obsessively. I have special soap that removes the gluten, I have my special mask. It’s so strict. But if my boss was to ever go, I would be stuck. She let’s me control everything, clean it all, have it my way.* **Debbie**  *I hadn’t really thought of the supermarket as a danger. But you’re both right, it is a “red zone.” Similar to [Hannah], I get worried about gluten in the house and the house becoming a danger zone.* **Fahdah** |
| Fear of gluten I can’t see | Being Near Gluten-Containing Food (3 participants)  Close vicinity to gluten containing food is seen as a potential source of contamination. | *We get food delive[ries] at work and some of it is gluten. I thought I’m going to have to stop my job. I can’t work if there’s food there. But I try not to let it stop me, so no eating at work. I only really eat at home, away from other food. Gluten free, cooked by me. It has made long shifts or work trips hard. I can no go a few days without if I need to, it’s very unfair.* **Michael**  *It’s the crumbs that worry me. You can see them on those conveyor belts at the checkout, you can see them on the floor. There are gluten specks everywhere, waiting.* **Hannah** |
|  | Airborne Gluten (4 participants)  Participants believe that contamination can occur due to respiratory ingestion of both food and non-food gluten-containing products | *The bread is unwrapped in those boxes, ready to be picked up. The aisle SMELLS of gluten. You can actually smell the bread. Now call me crazy, but if you smell the bread, the gluten must be in the air. And if the gluten is in the air, it can get into my body. Whether it’s through the lungs or the skin, it’s there.* **Michael**  *I took some time off work after I was diagnosed because I was worried about going back. The flour in the air, breathing it in.* **Debbie**  *But yeah gluten flour, can be airborne for over 24 hours if used in a kitchen and it settles likes dust on everything....we can inhale it and that tiny bit washes down with our mucus into our stomachs.* **Tiffany** |
|  | Contamination from Touching or Breathing in Non-Food Products (6 participants)  Participants have beliefs that skin contact or respiratory inhalation of gluten-containing non-food household products can lead to gluten cross-contamination. | *I don’t think it’s just about prepping food though. I don't know if my soap and hair products have gluten in them. I have really difficult to manage hair and Pantene is the only product that works in my hair. Seriously. I heard that if you wash your hands after using lotion and shampoo, its ok...But idk* [I don’t know] *about my soap.* **Hannah**  *So logically, I think I know I should be able to touch gluten. But that doesn’t comfort me. If I have touched gluten, and it’s on my hands, it can get into my mouth which = contamination.* **Michael** |
|  | Contamination from Animals (2 participants) | *My worst one was last week, I got cross-contaminated by non-human food! I was dog-sitting two dogs who get lots of treats. I started to get sick (and I KNOW I was eating gluten-free) and I figured out it was the dog treats. It's ridiculous!* **Debbie**  *Now, recently we’ve had a lot of crows around the surgery, so I can only assume that one of the birds picked up some bread, dropped it in the dog bowl, dog eats the bread and then infects me.* **Jade**  **She then says:** *So the children must have played with the dog, the wheat must have gotten on their hands, and then I got contaminated during our evening cuddles. Honestly, it’s so hard to stay away from all the risks.* |
|  | Contamination from Other People (5 participants)  People are seen as a source of cross-contamination This idea is extended to include all contact in particular that unlikely to result in cross-contamination. Such as sitting with others while eating safely prepared food.  Three participants believe that they might be cross-contaminated by kissing a partner who has ingested gluten. | *If you’ve got other people eating crumbs of gluten around you, that may contaminate my food. So I actually, I tend to eat alone at work. It’s very restricting. But it’s worth it because if people = contamination, it’s better to eat safely, and alone.* **Michael**  *No kissing hubby if he's "not safe"... I've gotten reactions from him sipping out of my cup when he's had something with gluten and then I drank out of it. Besides, better safe than sorry anyhow.* **Fahdah** |
|  | Contamination from the Self (1 participant)  Fears that she could self-contaminate by producing her own gluten internally. | *I read on the internet about this thing called the ketone diet, where basically you fast for several hours and it’s really good for weight loss. But I was reading that it can be dangerous for coeliacs because when your body produces ketones, it burns the fat muscles which contain gluten – so can you like internally gluten yourself?* **Tiffany** |
| Explanation of Routes to Cross-Contamination | Justification/Reasoning (7 participants)  A combination of gastro-intestinal symptoms, ill-reasoned science/medicine-based evidence and other people’s opinions. These appear to arise from the expressed opinions of friends, family, websites, social media, and internet support forums, rather than accurate clinical evidence or medical advice.  Medical evidence is evaluated as less credible due to differing medical opinions or research. | *But yeah gluten flour, can be airborne for over 24s hours if used in a kitchen and it settles likes dust on everything....we can inhale it and that tiny bit washes down with our muccous into our stomachs.* **Tiffany**  *I don't think I'm paranoid--it really does make me that sick!* **Tiffany**  *well, let's get real here---if it involves swapping spit--well, yes, it is a problem. Without getting too "icky"--there is mucosa in your mouth lining and swallowing ...etc, etc.* **Jade**  *I don’t think people know how hard it is and how awful it is and how DANGEROUS it is. I don’t think there’s enough medical knowledge about this. And that’s why different doctors say different things, because no one knows. I agree with you all though, we are talking from experience. We know how to do this.* **Poppy** |
|  | Certainty Around Implausible Beliefs (6 participants)  Resistant to Challenge | *I checked everything, step by step. The makeup was the clear culprit.* **Tiffany**  *It seems crazy but I’m sure it’s the only way.* **Jade**  **Debbie** describes the following incident: “*I was away with my friends, I think we had been in a hotel on the beach for a few days, a nice relaxing break. My lovely husband, he thought he’d treat me and redecorate the house. Now our walls are painted in the house. But my husband put up wallpaper, but he forgot that wallpaper paste has gluten in it. So the gluten is in the air and it’s in my home. I can’t escape it, it’s in that room. I don’t go in that room anymore. My mum thinks I’m crazy but luckily my husband understands. We don’t have gluten in the house, no gluten food, no gluten wallpaper paste. Entirely gluten free.”*  **Michael** responds “*But the wallpaper paste is glued to the wall? It can’t get into the air? So you shouldn’t be able to breathe it in? Unless you’re eating it?*”  **Debbie** replies “*I don’t know. To me it’s not safe. It is gluten. Gluten is bad.”* |
|  | Awareness (5 participants)  Participants appear to be aware that their ideas may not be well-reasoned and might also viewed by others as implausible. | *I have decided to become paranoid about gluten cross contamination. It's only my health, after all.* **Fahdah**  *It is probably a bit too “over the top” to worry about traces of gluten lurking on shop products.* **Michael** |
